# Paraganglioma of the pancreas: a potentially functional and malignant tumor

**DOI:** 10.1186/1477-7819-12-218

**Published:** 2014-07-17

**Authors:** Liyang Zhang, Quan Liao, Ya Hu, Yupei Zhao

**Affiliations:** 1 Department of General Surgery, Peking Union Medical College Hospital, Peking Union Medical College, Chinese Academy of Medical Sciences, No. 1, Shuaifu Garden, Dongcheng District, 100730 Beijing, China

**Keywords:** Pancreatic neuroendocrine tumor, Paraganglioma

## Abstract

Paragangliomas are neoplasms that arise from extra-adrenal chromaffin cells. Pancreatic paragangliomas are rare, and few are malignant. To the best of our knowledge, no cases of functional pancreatic paragangliomas have been reported in the literature to date. We present two cases of pancreatic paragangliomas with pathological confirmation. In the case 1, clinical testing and pathological analysis revealed functional and malignant characteristics of the tumor, which carried a poor prognosis. In case 2, functional paraganglioma was suspected. The clinical presentations and outcomes of these two patients are summarized, and the relevant literature is reviewed. Because of the small number of cases reported previously, few characteristics of these tumors are known. The best methods of predicting the malignant and functional potential of these tumors remain unknown. We propose careful preoperative treatment and close postoperative follow-up of paraganglioma patients because of the functional and malignant potential of these tumors.

## Background

Paragangliomas (PGLs) are rare neuroendocrine tumors (NETs) that arise from extra-adrenal chromaffin cells of the autonomic nervous system, with an average annual incidence rate of only 2 to 8 per 1 million adults [1]. Pancreatic PGLs are uncommon. Only 20 cases have been reported worldwide, and they have been limited to individual case reports in either radiography or pathology journals [2,3]. The case reports published to date contain no descriptions of functional pancreatic PGLs, and few cases were malignant [4]. We present two cases of pancreatic PGL. In one case, the clinical manifestation revealed a functional pancreatic PGL with liver metastases. In the other, functional pancreatic PGL was suspected. The clinical presentation and outcomes of the two cases are summarized, and we describe the functional and malignant potential of pancreatic PGLs based on our review of the literature.

## Case presentation

### Case 1

A 50-year-old woman presented to our institution with a 3-year history of intermittent hypertension, headache, sweating and palpitation. Abdominal computed tomography (CT) showed a 6-cm, solid, well-vascularized tumor on the head of the pancreas, with multiple liver metastases. Fine-needle aspiration (FNA) was performed. The cytopathological result indicated pancreatic PGL with typical double-positive chromogranin A(CgA) and synaptophysin (Syn) staining. The patient underwent laparotomy without special preoperative preparation. During the operation, the patient’s systolic blood pressure increased to 220 mmHg. Therefore, the operation was halted. Postoperative laboratory tests revealed the patient had a sixfold increase in 24-hour urinary norepinephrine excretion, consistent with functional pancreatic PGL. Chemotherapy with 5-fluorouracil, mitomycin and doxorubicin was administered postoperatively without significant remission. The patient died 4 years after her initial diagnosis.

### Case 2

A 63-year-old man with a 10-year history of hypertension was admitted to the hospital for treatment of a suspected functional pancreatic PGL, although symptoms of catecholamine excess, such as headache and palpitation, were not present. Abdominal CT revealed a 4-cm, solid, well-vascularized tumor on the head of the pancreas and adjacent to the superior mesentery vein. ^123^I-metaiodobenzylguanidine (^123^I-MIBG) imaging revealed abnormal uptake in the region of the pancreatic head. Although the patient had a normal level of 24-hour urinary norepinephrine excretion, primary functional pancreatic PGL was suspected due to his hypertension history. As a result, α-adrenergic receptor blockers were administered for 2 weeks prior to surgery. During surgery, the patient’s blood pressure remained stable, and the tumor was resected successfully. Pathological analysis confirmed the diagnosis of pancreatic PGL, and the tumor was positive for CgA and Syn, observed by performing immunohistochemical analysis. The patient received no subsequent adjuvant treatment, and his blood pressure was normal 3 months after surgery.

## Discussion

Primary pancreatic PGL is rare, with only 20 cases reported in the literature to date. Similar to other pancreatic NETs, they are more likely to affect women than men older than 40 years of age. Patients typically present with abdominal pain or are asymptomatic, and PGL is discovered incidentally on routine radiographic images. Pancreatic PGLs can arise in any part of the pancreas, although the majority of these tumors are located at the head of the pancreas [4]. The size of the tumors ranges from 3 to 20 cm. Larger tumors are often accompanied by cystic changes. The location of pancreatic PGLs can usually be identified by using abdominal CT or MRI, which have high sensitivity for PGL. Functional ^123^I-MIBG scans often provide accurate information on the disease extent and biology [5], especially in malignant cases, because of high uptake in chromaffin cells. PGL cases are typically hypervascular, with cystic changes mimicking most pancreatic NETs. Therefore, definitive diagnosis requires pathological confirmation. FNA can be performed preoperatively, but diagnostic accuracy is low [4]. Immunohistochemical analysis is necessary to confirm the presence of the characteristic CgA-Syn double-positive cell populations [4].

PGLs rarely produce excess catecholamines [6], although patients with functional PGL may have symptoms of catecholamine excess, such as hypertension, headache and palpitation. Ultimately, the diagnosis of functional pancreatic PGL is made on the basis of the levels of catecholamines and their metabolites in plasma or urine. To the best of our knowledge, no functional pancreatic PGLs have been reported previously. Herein, we present a patient with typical PGL symptoms and a sixfold increase in 24-hour urinary norepinephrine excretion, consistent with the findings of a functional PGL. Because the patient’s norepinephrine levels were not known preoperatively, the surgery was performed without preoperative α-adrenergic receptor blockade treatment, which resulted in unstable blood pressure that necessitated halting the surgery. In the second case, as the patient was suspected with functional pancreatic PGL, he received α-adrenergic receptor blocker treatment 2 weeks prior to the operation in order to avoid the excess release of catecholamine.

PGLs are more likely to be malignant (29% 40%) [6]. Malignant PGLs are defined as those that metastasize, recur or show evidence of local invasion [7]. Because of the poor prognosis of malignant PGL, it is important to distinguish between benign and malignant tumors before surgery. Frequently, PGLs may develop metastases years after the initial diagnosis [8], making early identification of metastasis critical to improving outcomes. Unfortunately, no reliable genetic, molecular or imaging markers currently exist to predict the malignant potential of PGLs.

A multimodal approach in the treatment of patients with PGL is mandatory, including advanced surgery, nuclear medicine, chemotherapy, radiotherapy and novel biologically targeted drugs. Traditionally, treatment for all PGLs has begun with complete surgical excision. In addition, minimally invasive surgical techniques and close cooperation with the anesthetist are critical to minimizing intraoperative fluctuation of blood pressure. Treatment with high-dose ^131^I-MIBG in patients with PGLs with metastases may often result in sustained remission or stable disease [9]. Similarly, treatment with radiolabeled somatostatin receptor ligands may benefit patients with high uptake observed on octreotide scintigraphy images [10].

## Conclusion

We present two cases of functional pancreatic PGLs. Pancreatic PGLs have functional and malignant potential requiring careful preoperative preparation, and close postoperative follow-up should be considered for optimal patient outcomes.

## Consent

Written informed consent was obtained from the patients or patient`s family members for publication of this case. A copy of the written consents is available for review by the Editor-in-Chief of this journal.

## Abbreviations

CgA: Chromogranin A; CT: Computed tomography; FNA: Fine-needle aspiration; I-MIBG: ^123^I-Metaiodobenzylguanidine; NET: Neuroendocrine tumor; PGL: Paraganglioma; Syn: Synaptophysin.

## Competing interests

The authors declare that they have no competing interests.

## Authors’ contributions

All authors were involved in the care of the patients. All authors read and approved the final manuscript.
